# Supplementary material for: Keratin 14-dependent disulfides regulate epidermal homeostasis and barrier function via 14-3-3σ and YAP1
Source: eLife. 2020 May 5;9:e53165. doi: 10.7554/eLife.53165 (PMC7250575; doi:10.7554/eLife.53165)
Supplement: Supplementary file 4. [file elife-53165-supp4.docx]

| **Supplementary Table 4: Key Resources Table** (Guo et al.) | | | | |
| --- | --- | --- | --- | --- |
| List of abbreviations used in this table:  WT: wildtype  HA: hemagglutinin  STR: short tandem repeat  CF: cysteine free  GFP: green fluorescent protein  IHC: immunohistochemistry  IF: immunofluorescence  WB: Western blot  IP: immunoprecipitation  PLA: proximity ligation assay  Cat. #: catalog number  N/A: not applicable  PCR: polymerase chain reaction  EdU: 5-ethynyl-2-deoxyuridine  TUNEL: terminal deoxynucleotidyl transferase mediated dUTP nick end labelling  TCEP: tris(2-carboxyethyl)phosphine  DAPI:  4,6-diamidino-2-phenylindole | | | | |
| **Reagent type (species) or resource** | **Designation** | **Source or reference** | **Identifiers** | **Additional information** |
| Genetic reagent (Mus. musculus) | C57BL/6J | Jackson Laboratory |  |  |
| Genetic reagent (*Mus. musculus*) | *Krt14* C373A | This paper |  | New mouse line (C57BL/6J) with a knock-in Cys->Ala mutation at codon 373 in the *Krt14* gene |
| Genetic reagent (Mus. musculus) | *Krt14^-/-^* | Feng and Coulombe, 2015 |  | *Krt14* null mice |
| Cell in primary culture (*Mus. musculus*) | skin keratinocytes | This paper |  | Skin keratinocytes isolated from newborn *Krt14* C373A mice (and WT littermates controls) and seeded in primary culture |
| cell line (human) | HeLa (cervical adenocarcinoma) | ATCC |  | Authenticated ATCC STR profiling |
| transfected  construct (*M. musculus*) | HA-14-3-3σ | Addgene | Plasmid #11946 |  |
| transfected construct (human) | GFP-K14 WT | Feng and Coulombe, 2015 |  |  |
| transfected construct (human) | GFP-K14 CF | Feng and Coulombe, 2015 |  |  |
| transfected construct (human) | GFP-K14 C376A | Feng and Coulombe, 2015 |  |  |
| transfected construct (human) | GFP-K14 CF-C367 | Feng and Coulombe, 2015 |  |  |
| antibody | Anti-K14 (chicken polyclonal) | Biolegend | Cat. #: 906001 | IHC (1:1000)  IF (1:100) |
| antibody | Anti-K14 (rabbit polyclonal) | Biolegend | Cat. #: 905301 | WB (1:100)  IHC (1:100)  IF (1:100)  IP (1:100)  PLA (1:100) |
| antibody | Anti-K14 (mouse monoclonal) | Abcam | Cat. #: ab7800 | IF (1:100)  PLA (1:100) |
| antibody | Anti-K5 (rabbit polyclonal) | Biolegend | Cat. #: 905504 | IHC (1:500)  IF (1:500)  WB (1:500) |
| antibody | Anti-β-actin (mouse monoclonal) | Sigma-Aldrich | Cat. #: A5441 | WB (1:5000) |
| antibody | Anti-K10 (rabbit polyclonal) | Biolegend | Cat. #: 905401 | IHC (1:1000) |
| antibody | Anti-Filaggrin (rabbit polyclonal) | Biolegend | Cat. #: 905801 | IHC (1:500)  WB (1:1000) |
| antibody | Anti-Loricrin (rabbit polyclonal) | Biolegend | Cat. #: 905101 | IHC (1:1000)  WB (1:1000) |
| antibody | Anti-14-3-3σ (goat polyclonal) | Santa Cruz Biotechnology | Cat. #: sc-7683 | IHC (1:100)  WB (1:500)  IF (1:100) |
| antibody | Anti-14-3-3σ (rabbit polyclonal) | Sigma-Aldrich | Cat. #: PLA0201 | IF (1:2000) |
| antibody | Anti-YAP (mouse monoclonal) | Santa Cruz Biotechnology | Cat. #: sc-101199 | IHC (1:100)  IF (1:100)  WB (1:500)  PLA (1:100) |
| antibody | Anti- Phospho-YAP (rabbit polyclonal) | Cell Signaling Technology | Cat. #: 4911 | WB (1:1000) |
| antibody | Anti-YAP (rabbit polyclonal) | Cell Signaling Technology | Cat. #: 4912 | WB (1:1000) |
| antibody | Anti-HA (mouse monoclonal) | Thermo Fisher Scientific | Cat. #: 26183 | IF (1:200)  WB (1:1000) |
| antibody | Anti-HA (rabbit polyclonal) | Sigma-Aldrich | Cat. #: H6908 | WB (1:1000) |
| antibody | Anti-α-E-Catenin (rabbit polyclonal) | Cell Signaling Technology | Cat. #: 3236S | IHC (1:200)  IF (1:200) |
| antibody | Anti-Lamin A/C (goat polyclonal) | Santa Cruz Biotechnology | Cat. #: sc-6215 | IHC (1:100)  IF (1:100) |
| antibody | Anti-Desmoglein 1 (mouse monoclonal) | Progen | Cat. #: 61002 | IHC (1:100)  IF (1:100) |
| antibody | Anti-Phospho-MLC2 (rabbit polyclonal) | Cell Signaling Technology | Cat. #: 3671S | IF (1:50) |
| antibody | Anti-Vinculin (mouse monoclonal) | Millipore | Cat. #: MAB3574 | IF (1:200) |
| antibody | Anti-E-cadherin (rabbit monoclonal) | Cell Signaling Technology | Cat. #: 3195S | IHC (1:200) |
| antibody | Anti-Claudin 3 (rabbit polyclonal) | Thermo Fisher Scientific | Cat. #: 34-1700 | IHC (1:100) |
| antibody | Anti-Desmoplakin (rabbit polyclonal) | K. Green, Northwestern University, Evanston, IL | N/A | IHC (1:100) |
| antibody | Anti- Alpha-catenin a-18 (rat monoclonal) | Nagafuchi A., Nara Medical University, Kashihara, Nara, Japan | N/A | IHC (1:500) |
| antibody | Anti- p63-α (D2K8X) XP^®^ (rabbit monoclonal) | Cell Signaling Technology | Cat. #13109 | IHC (1:200) |
| antibody | Alexa Fluor 488 Goat Anti-Mouse IgG (H+L) | Thermo Fisher Scientific | Cat. #: A-11001 | IF (1:500) |
| antibody | Alexa Fluor 488 Goat Anti-Rabbit IgG (H+L) | Thermo Fisher Scientific | Cat. #: A-11008 | IF (1:500) |
| antibody | Alexa Fluor 546 Goat Anti-Rabbit IgG (H+L) | Thermo Fisher Scientific | Cat. #: A-11010 | IF (1:500) |
| antibody | Alexa Fluor 555 Donkey Anti-Mouse IgG (H+L) | Thermo Fisher Scientific | Cat. #: A-31570 | IF (1:500) |
| antibody | Alexa Fluor 488 Goat anti chicken IgY (H+L) | Thermo Fisher Scientific | Cat. #: A11039 | IF (1:500) |
| sequenced-based reagent | Krt14 WT_F | This paper | genotyping PCR primers | 5’-AGACCAAAGGCCGTTACTG-3’ |
| sequenced-based reagent | Krt14 WT_R | This paper | genotyping PCR primers | 5’-TTGAGGTGGAGGAGGAGTCT-3’ |
| sequenced-based reagent | Krt14 C373A _F | This paper | genotyping PCR primers | 5’-ACCAAAGGCCGTTACGC-3’ |
| sequenced-based reagent | Krt14 C373A_R | This paper | genotyping PCR primers | 5’-GAAGCCAAGTCACACCCCTG-3’ |
| commercial assay or kit | Click-iT Plus EdU Alexa Fluor 488 Imaging Kit | Thermo Fisher Scientific | Cat.#: C10637 |  |
| commercial assay or kit | EDU | Thermo Fisher Scientific | Cat.#: A10044 |  |
| commercial assay or kit | TUNEL enzyme | Roche applied Science | Cat. #: 11767305001 |  |
| commercial assay or kit | TUNEL label mix | Roche applied Science | Cat. #: 11767291910 |  |
| commercial assay or kit | Duolink® In Situ Red Starter Kit Mouse/Rabbit | Sigma-Aldrich | Cat. #: DUO92101 |  |
| commercial assay or kit | P1 Primary Cell 4D-Nucleofector™ X Kit | Lonza | Cat. #: V4XP-1024 |  |
| commercial assay or kit | SE Cell Line 4D-Nucleofector™ X Kit S | Lonza | Cat.#: V4XC-1032 |  |
| commercial assay or kit | Dual-Glo® Luciferase Assay System | Promega | Cat. #: E2920 |  |
| chemical compound, drug | Acetone | Sigma-Aldrich | Cat. #: 34850 |  |
| chemical compound, drug | Empigen BB detergent | Sigma-Aldrich | Cat. #: 30326 |  |
| chemical compound, drug | TCEP solution | Thermo Fisher Scientific | Cat. #: 77720 | chemical compound, drug |
| Software, algorithm | ImageJ software | ImageJ (<http://imagej.nih.gov/ij/>) |  |  |
| Software, algorithm | ZEN lite 2.6 | ZEISS (<https://www.zeiss.com/microscopy/us/products/microscope-software/zen-lite.html>) |  |  |
| Software, algorithm | GraphPad Prism 8 | GraphPad Prism ([https://graphpad.com](https://graphpad.com/)) |  |  |
| other | DAPI stain | Thermo Fisher Scientific | Cat. #: D1306 | (1 µg/mL) |
| other | TEWAMETER | Courage and Khazaka | Cat. #: TM300 |  |
| other | Protein G Sepharose | GE healthcare | Cat. #: 17-0618-01 |  |
| other | Alexa Fluor 488 Phalloidin | Thermo Fisher Scientific | Cat. #: A123791 |  |
